# Bioinspired DNA Nanointerface with Anisotropic Aptamers for Accurate Capture of Circulating Tumor Cells

**DOI:** 10.1002/advs.202000647

**Published:** 2020-08-09

**Authors:** Weiwei Qin, Liang Chen, Zhiru Wang, Qian Li, Chunhai Fan, Minhao Wu, Yuanqing Zhang

**Affiliations:** ^1^ Guangdong Key Laboratory of Chiral Molecule and Drug Discovery School of Pharmaceutical Sciences Sun Yat‐sen University Guangzhou Guangdong 510006 China; ^2^ College of Materials and Energy South China Agricultural University Guangzhou Guangdong 510642 China; ^3^ State Key Laboratory of Chemo/Biosensing and Chemometrics Hunan University Changsha 410082 China; ^4^ School of Chemistry and Chemical Engineering Shanghai Jiao Tong University Shanghai 200240 China; ^5^ Zhongshan School of Medicine Sun Yat‐sen University Guangzhou 510080 China

**Keywords:** aptamers, cell capture, circulating tumor cells, DNA, nanointerfaces

## Abstract

The capture and analysis of circulating tumor cells (CTCs) have provided a non‐invasive entry for cancer diagnosis and disease monitoring. Despite recent development in affinity‐based CTCs isolation, it remains challenging to achieve efficient capture toward CTCs with dynamic surface expression. Enlightened by the synergistic effect insideimmune synapses, the development of a nanointerface engineered with topology‐defined anisotropic aptamers programmed by DNA scaffold (DNA nanosynapse), for accurate CTCs isolation, is herein reported. As compared to isotropic aptamers, the DNA nanosynapse exhibits enhanced anchoring on the cell membrane with both high and low epithelial cell adhesion molecule (EpCAM) expression. This nanointerface enables accurate capture toward CTCs of heterogeneous EpCAM, without dramatically proportional change inside the mixture of diverse phenotypes. By applying this nanoplatform, CTCs detection as well as downstream analysis for measuring disease status can be achieved in clinical samples from breast cancer patients.

## Graphical abstract

Inspired by the synergistic effect inside immune synapses, a nanointerface engineered with topology‐defined anisotropic aptamers programmed by DNA scaffold is developed through thermal annealing. Such a nanointerface enables enhanced membrane anchoring and accurate capture toward CTCs of heterogeneous EpCAM, without dramatically proportional change inside the mixture of diverse phenotypes. This nanoplatform can be applied for CTCs detection in clinical samples.

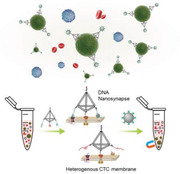

## 1 Introduction

Circulating tumor cells (CTCs) possess information about a tumor, and the analysis of CTCs are critical to cancer diagnosis, treatment guidance, as well as prognosis.^[^ ^1^, ^2^, ^3^, ^4^ ^]^ CTCs isolation, however, often suffer from its extreme rarity (several CTCs against billions of healthy blood cells in the circulation).^[^ ^5^ ^]^ Current strategies for CTCs enrichment can be categorized into antigen‐dependent isolation and label‐free isolation (cell size, deformability, or density).^[^ ^6^, ^7^, ^8^ ^]^ Among these methods, DNA aptamers have shown great promise as capture ligand for CTCs, with properties superior to antibodies, including low cost, high storage stability, small size, and reproducible quality.^[^ ^9^, ^10^, ^11^, ^12^ ^]^ Despite its advantages, the binding affinity of aptamer are likely to be compromised in a complicated condition especially real blood sample, hindering its translation to clinical setting.

Multivalent display offers a powerful way to tune and enhance the binding affinity between target receptors and weakly capture ligands.^[^ ^13^, ^14^, ^15^ ^]^ Several groups have demonstrated the engineering of multivalent aptamer for a lower dissociation constant, giving rise to highly efficient CTCs capture.^[^ ^16^, ^17^, ^18^, ^19^, ^20^, ^21^ ^]^ As a recent example, one study has taken advantage of DNA framework to control the spatial organization of trivalent aptamers against EpCAM (epithelial cell adhesion molecule). Such topological engineering not only merely increases the ligand binding affinity with the membrane receptors, but also prevents the aptamer from endocytosis by cells, which has been evaluated by improved CTCs capture. Unfortunately, the expression of EpCAM and other surface markers on CTCs is inevitably heterogeneous in real samples.^[^ ^22^, ^23^ ^]^ For instance, those CTCs undergoing epithelial to mesenchymal transition (EMT) during tumor progression, would exhibit downregulated EpCAM expression. In this context, homo‐ligand‐based isolation, such as FDA‐cleared CellSearch, is hampered by low efficacy when it comes to post‐EMT CTCs. Hence, the information reflecting disease progress would be lost in a certain extent, when relying on unitary aptamers for CTC capture.

**Scheme 1:**
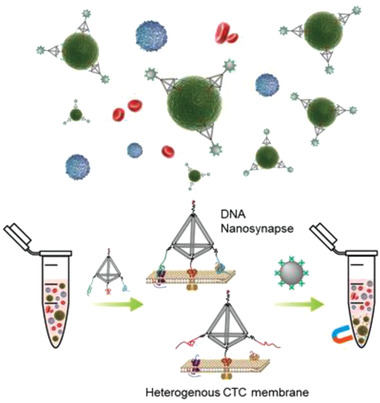
Overview of the DNA nanosynapse for highly efficient capture of circulating tumor cells.

To address the above issue, many methods based on cocktail of various capture ligand had been exploited for the accurate capture of CTCs.^[^ ^24^, ^25^ ^]^ To date, the successful improvement of capturing heterogeneous CTCs via cocktail of aptamers was achieved by decorating different aptamers on the same substrates.^[^ ^26^, ^27^ ^]^ However, the conformation of aptamers would be affected by the interaction between substrates and aptamers, leading to limited capture performance.^[^ ^28^ ^]^ In immunology, recruitment of different receptors has been existed at the interface between two immune cells.^[^ ^29^, ^30^ ^]^ These naturally evolved membrane structures are known as immunological synapse, where the cooperative binding of distinct receptors contributes to a maximal cell–cell interaction.^[^ ^31^ ^]^ Herein, enlightened by these synergy effect adopted by the immune system, we developed a nanointerface composed of topology‐defined anisotropic ligands programmed by DNA scaffold, termed DNA nanosynapse, in order to accurately capture the heterogeneous CTCs from breast cancer. To construct the nanosynapse, we select tetrahedral DNA nanostructures (TDNs) as the scaffolds with its defined valence, extraordinary structural stiffness, and stability.^[^ ^32^, ^33^ ^]^ Distinct aptamers are couple to three vertices of the same DNA nanostructure in a coplanar manner, forming the interface to interact with cell membrane (**Scheme** **1**). In contrast to other nanoplatforms, the developed nanointerface could be easily fabricated by simple annealing, without complex chemical modification on substrates. By employing hetero‐multivalent aptamers, the nanosynapse could enduringly adhere on the membrane of CTCs with distinct EpCAM expression profile, instead of endocytosis by the cells, which is an important limiting factor for CTCs capture. With its excellent capture of reduced EpCAM‐expressing cells, the DNA nanosynapse is expected to offer a highly efficient and reliable strategy for CTCs detection.

## 2 Results and Discussion

### 2.1 Construction and Cellular Distribution of the DNA Nanosynapse

**Figure 1:**
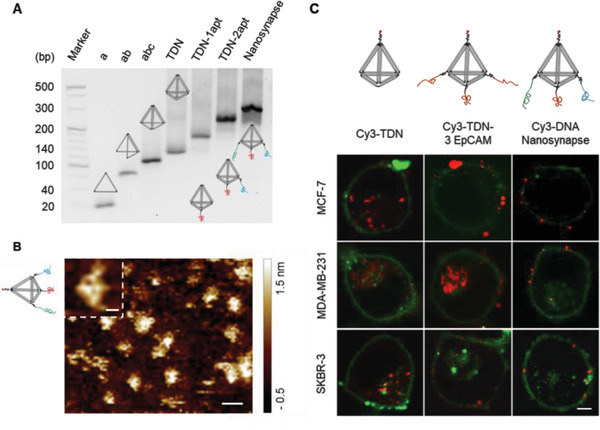
Assembly and cellular distribution of the DNA nanosynapse. A) Agarose electrophoresis of the folded DNA nanostructure. B) AFM image of the assemble DNA nanostructures. Scale bar: 20 nm; Inset: 5 nm. C) CLSM of three different cell lines incubated with TDN, TDN‐3 EpCAM, and DNA nanosynapse respectively for 4 h. The nanostructures were labeled with Cy3 (Red) and cell membrane was stained with DiO (green). Scale bar: 5 µm.

To prepare the DNA nanosynapse, four scaffold oligonucleotides forming the tetrahedral DNA nanostructures (TDNs) were annealed, together with three chosen aptamer strands targeting surface protein EpCAM, HER2, and EGFR, respectively (details about the assembly of TDNs are shown in Figure S1 and Table S1, Supporting Information).^[^ ^9^, ^34^ ^]^ The DNA nanosynapse was designed to contain framework edges of 17 base pair and three linkers of 20 base pair conjugated between the scaffold vertices and aptamers. The successful formation of DNA nanosynapse was first verified by increasingly slow mobility of a single band following the sequential addition of single‐strand DNA in agarose electrophoresis (yield > 78.5%, **Figure** **1**A; Figure S2, Supporting Information). In addition, the programmed structure of DNA nanosynapse with pyramidal configuration was imaged by atomic force microscopy (AFM) (Figure 1B).

The effective anchoring of capture ligand on target cell membrane is a significant element for CTCs capture. As the DNA nanosynapse can be considered as nanoparticles in the field of delivery system, it is likely to encounter internalization, and thus the ligand presentation on cell membrane could be hampered.^[^ ^35^, ^36^, ^37^ ^]^ In this regard, we next studied the cellular distribution of the DNA nanosynapse on different cell lines with distinct surface profile, in order to mimic the heterogeneous CTCs in real sample. MCF‐7, SKBR‐3, and MDA‐MB‐231 cell lines were chosen in the view of their well characterized EpCAM expression level, which were high to low respectively.^[^ ^38^, ^39^, ^40^ ^]^ To visualize the membrane‐anchor or cell‐endocytosis event, DNA scaffold and cell membrane were labeled with cy3 fluorophore and 3,3′‐Dioctadecyloxacarbocyanine Perchlorate (DiO), respectively. With regard to MDA‐MB‐231 cells, the cell line was characterized with low EpCAM, HER2, as well as EGFR expression.^[^ ^39^, ^41^ ^]^ Therefore, we first selected EpCAM aptamers as a representative to construct homo multivalent nanointerface which have been reported previously, other than the cocktail of different isotropic aptamer.^[^ ^42^ ^]^ Hence, we also assembled and examined TDNs without aptamers, as well as TDNs conjugated with three EpCAM aptamers (TDN‐3 EpCAM). As observed in confocal laser scanning microscopy (CLSM), the DNA nanosynapse showed firm membrane adhesion on MCF‐7, SKBR‐3, and MDA‐MB‐231 cell lines (Figure 1C). With the removal of binding ligand, bare TDNs were inherently endocytosed by all three cell lines.^[^ ^37^ ^]^ Furthermore, a mixture of TDNs coupled with only one aptamer toward different receptor, would also encounter cellular internalization due to their low binding affinity (Figure S3, Supporting Information). In the meantime, TDN‐3 EpCAM bound predominated on membrane of relatively high EpCAM cells (MCF‐7 and SKBR‐3), which was in accord with previously study. On the contrary, TDN‐3 EpCAM was internalized by low‐expressing cells (MDA‐MB‐231). The enhanced cell adhesion of DNA nanosynapse toward low EpCAM cell lines is hypothetically owing to the synergetic recruitment of different receptors on cell membrane by topology‐defined anisotropic ligands. Next, we used CLSM to evaluate whether these receptors could recruit synergistically on cell membrane, and thus allow DNA nanosynapse bind to these proteins at the same cell.^[^ ^43^ ^]^ MDA‐MB‐231 cells were first incubated with aptamers, which targeted two different proteins and were conjugated with Cy5 and Cy3, respectively. After treatment with locking strands that could brought two aptamers together with same size as DNA nanosynapse (two aptamers spacing 17 bp), fluorescence resonance energy transfer (FRET)‐induced Cy5 fluorescence signal was observed on the cell membrane (Figure S4A‐D, Supporting Information). We also performed acceptor photobleaching to further verify the FRET process. After the Cy5 was photobleached with 640 nm laser, the emission of Cy3 was enhanced (Figure S4E‐G, Supporting Information). These results indicated that these proteins could recruit together and further allow DNA nanosynapse to recognize synergistically.

### 2.2 Magnetic Capture toward Heterogeneous Cells

**Figure 2:**
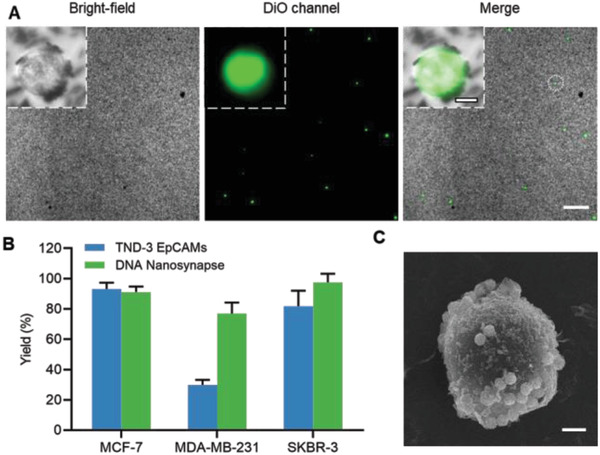
Enhanced capture toward cell with heterogeneous surface expression by the DNA nanosynapse. A) Fluorescence microscope images of the captured MCF‐7 cells labeled with DiO (green). Insets are zoom‐in images from the dotted circle. Scale bar: 200 µm; Inset: 5 µm. B) Comparison of capture efficiency between TND‐3 EpCAMs and DNA nanosynapse. C) SEM image of MCF‐7 cells captured by DNA nanosynapse with conjugated magnetic beads. Scale bar: 2 µm.

Having validated the enhanced membrane anchoring DNA nanosynapse, we then applied the nanoplatform for CTCs capture. We first evaluated the capture efficiency of two different strategies (Figure S5A, Supporting Information). In the single‐step strategy, the DNA nanostructures were conjugated to the magnetic beads prior to incubation with cells. While in two‐step strategy, the DNA nanostructures and magnetic beads were incubated with cells successively. As shown in Figure S5B, Supporting Information, the two‐step strategy exhibited overwhelming cell capture efficiency (yield = 93.1%), when compared with single‐step strategy (yield = 64.2%). This could be contribution from the assurance of the preferential conformation of aptamers for target binding in two‐step strategy compared to single‐step strategy, prior to addition of magnetic beads.^[^ ^28^ ^]^ In all following studies, two‐step strategy was adopted for magnetic cell capture. To investigate the function of topology‐defined anisotropic ligands on capturing low EpCAM expressed cell, we compared the capture efficiency of DNA nanosynapse and TDN‐3 EpCAM. We spiked three different cell lines into phosphate buffered saline (PBS) respectively and then DNA nanosynapse or TDN‐3 EpCAM were incubated with cells followed by magnetic isolation. Captured cells were observed and counted by fluorescence microscope, with magnetic beads conjugated on the cell surface (**Figure** **2**A; Figure S6, Supporting Information). Furthermore, scanning electron microscope (SEM) verified the successful anchoring of magnetic beads on cell membrane (Figure 2C). The DNA nanosynapse and TDN‐3 EpCAM showed a comparable capture efficiency toward relatively high EpCAM expressed cells (MCF‐7 and SKBR‐3). This could be due to the accelerated initiation of multivalent binding between DNA nanosynapse and cell membrane by the abundant EpCAM in these two cell lines. Additionally, the dimerization of HER2 and EGFR would also contribute to multivalent binding.^[^ ^44^ ^]^ As such, appropriate amount of capture ligand would also be presented on the cell membrane in terms of DNA nanosynapse to ensure enough magnetic force executing cell capture.^[^ ^45^ ^]^ It is worth noting that a higher yield was observed for low EpCAM‐expressed cells (MDA‐MB‐231) captured by DNA nanosynapse (≈77.0%), compared to topology‐defined isotropous aptamers (≈29.8%) and other EpCAM‐based methods (Figure 2B; Table S2, Supporting Information).^[^ ^25^, ^45^, ^46^, ^47^ ^]^ However, previously published work by Zuo et al. reported a 75% capture efficiency toward MDA‐MB‐231 cells using TDN‐3 EpCAM, which was much higher than what we had observed (Table S3, Supporting Information).^[^ ^42^ ^]^ This was attributed to the differences in experiment procedure where we co‐spiked cells with 10^5^ Jurkat cells in PBS, rather than pure PBS adopted by previous work. We also compared the capture performance of DNA nanosynapse with that of the cocktail of TDN‐3 EpCAM, TDN‐3 EGFR, and TDN‐3 HER2, toward MDA‐MB‐231 cells, which were characterized with low EpCAM, low HER2, as well as low EGFR. The DNA nanosynapse showed a better capture performance, compared to the cocktail of isotropous TDN‐3 composed by three distinct aptamers (≈51.6%, Figure S7, Supporting Information). These data further revealed the enhanced anchoring of DNA nanosynapse on cell membrane with low protein expression.

**Figure 3:**
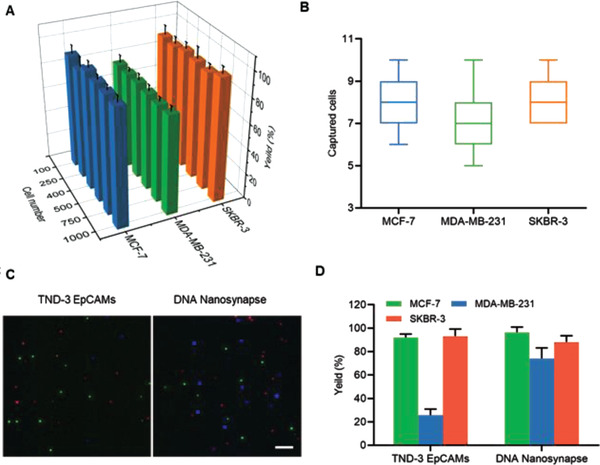
Capture of spiked cell in buffer. Cell enrichment efficiency toward different cell lines of A) 100–1000 or B) 10 cells by DNA nanosynapse. C) Fluorescent microscopy images of the captured cell mixture composed of MCF‐7 (DiO stained, green), MDA‐MB‐231 (Hoechest 33 342 stained, blue), and SKBR‐3 cells (Dil stained, red) in equal proportion. Scale bar: 200 µm. D) Capture efficiency of cell mixture same as (C) by DNA nanosynapse and TND‐3 EpCAMs.

We next proceed to evaluate the enrichment performance of DNA nanosynapse in a series of artificial CTCs samples. Various concentration of DiO‐stained cells (ranging from 100 to 1000 cells mL^−1^) were first spiked into PBS and then magnetic isolated through DNA nanosynapse. As shown in **Figure** **3**A,B, the DNA nanosynapse exhibits a high capture efficiency toward both EpCAM‐high cells (MCF‐7 and SKBR‐3) and EpCAM‐low cells (MDA‐MB‐231). Moreover, the average enrichment yield for 10 spiked cells of different phenotype in 1 mL solution reached above 70% (Figure 3B). We also examined cell capture under physiological condition by spiking the cells in human whole blood samples from healthy donors. The results indicated that the DNA nanosynapse retained its high capture efficiency (88%, 76%, and 91% for MCF‐7, MDA‐MB‐231, and SKBR‐3 cells, respectively), which was comparable to those carried out in PBS. Meanwhile, a low capture efficiency (≈0.28%) toward white blood cells was observed during CTCs isolation, indicating the high specificity of our nanoplatform under physiological condition (Figure S8, Supporting Information).

We further carried out capture experiment toward artificial hetero‐EpCAM‐expressed CTCs made of equal amount of MCF‐7, MDA‐MB‐231, and SKBR‐3 cells. These three cell lines were pre‐stained with different dyes as indicated in Figure 3 caption. Compared to TDN‐3 EpCAM, the DNA nanosynapse showed a more accurate cell capture, that the isolated cells were characterized with a more approximately 1:1:1 constitution (Figure 3C,D). In contrast, cells captured via TDN‐3 EpCAM exhibited a ratio imbalance in which high EpCAM cells (MCF‐7 and SKBR‐3 cells) accounted for a larger proportion.

Captured cells with high viability, as well as the release of CTCs are critical for follow‐up analysis, especially for disease monitoring, treatment identification, or 3D organoid generation. We captured the above spiked cell lines and treated with live cell staining dye Calcein‐AM. High cell viability was observed in all captured cell lines (Figure S9–11A, Supporting Information). Subsequent culture of the captured cell lines showed the maintaining of normal cellular morphology and self‐proliferation during a 7‐day observation (Figure S9–11B, Supporting Information). To achieve cell release, we further utilized Deoxyribonuclease I (DNase I) to destroy the junction composed of DNA nanostructure between the cell membrane and magnetic beads. After the enzymatic digestion, more than half of the captured cells were released regarding MCF‐7, MDA‐MB‐231, and SKBR‐3 cells. Moreover, the released cells exhibited high viability when subjected to live cell staining (Figure S12, Supporting Information).

### 2.3 CTC Isolation and Analysis from Clinical Samples

**Figure 4:**
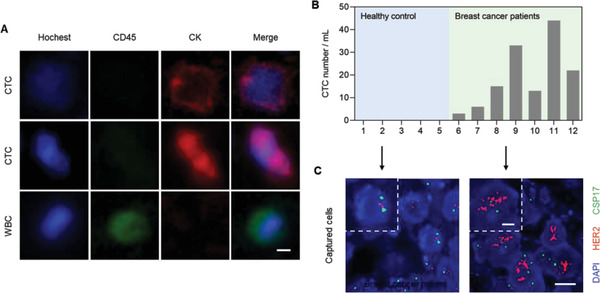
CTC detection and genetic analysis in patient sample. A) Fluorescence microscope images of captured CTCs and WBCs by DNA nanosynapse after staining with Hoechst (blue), cytokeratin (red), and CD 45 (green). Hoechst 33 342^+^/CD45^−^/CK^+^ cells with proper size were counted as CTCs. Scale bar: 5 µm. B) Quantification of the isolated CTCs from 0.5 mL blood sample from healthy donors (1–5) and breast cancer patients (6–12). C) FISH analysis of the captured cells. Cells were hybridized with CSP17 (green) and HER‐2 (red) probe. HER‐2 amplification appeared in isolated CTCs from patient with a HER‐2/CSP 17 ratio of greater than or equal to 2 (normal cells from healthy donors featured a ratio less than 2). Insets are zoom‐in images representing the arrow‐pointing areas. Scale bar: 5 µm; Inset: 2 µm.

Based on the above results, we next tested the potential of DNA nanosynapse for CTCs isolation in clinical setting. The whole blood samples were received from the First Affiliated Hospital of Sun Yat‐Sen University with informed consent. Blood samples from two healthy donors and six breast cancer patients were used for evaluation. CTCs isolated from samples were identified via cell morphology and common three‐color immunocytochemistry consisted of anti‐CD45 for white blood cells (WBCs), anti‐cytokeratin for epithelial cells, and Hoechst for nucleus. CTCs were characterized with proper cell size, intact nuclei, CD45 negative, and positive for DAPI as well as Cytokeratin (CK). On the other hand, cells stained positive for DAPI and CD45, while negative for CK were considered as WBCs (**Figure** **4**A). The number of CTCs isolated from seven breast cancer patients ranged from 3 to 44 cells per mL, with an average purity of 28.4%. Meanwhile, no signs of CTCs were detected in samples from five healthy donors (Figure 4B; Figure S13, Supporting Information).

To explore whether our system is compatible with common gene analysis for measuring disease status, samples from two of patients with CTC counts more than 20 cells per mL were then analyzed by fluorescence in situ hybridization (FISH). We measured the target gene Her2 by a specific probe and used centromere‐specific probe 17 (CSP17) as a control. HER‐2 amplification was detected in patients with a HER‐2/CSP 17 ratio of great than or equal to 2, and representative results were shown in Figure 4C, while no positive signal was detected in normal cells isolated from healthy donors.

## 3 Conclusion

To sum up, we have shown a bioinspired nanoplatform for precise capture of heterogeneous CTCs. The topology‐defined anisotropic aptamer (DNA nanosynapse) has provided an effective strategy to improve ligand anchoring on the surface of high EpCAM cells as well as relatively low‐expressing cells. Future studies addressing the receptor recruitment that underlie the binding event will further promote our understanding on how the DNA nanosynapse enhanced ligand adhesion. In addition, the DNA nanosynapse have shown to be used for CTCs detection toward breast cancer in clinical setting. Furthermore, owing to the modular design of DNA nanostructure, the developed nanoplatform could allow tuning the size of nanointerface and kinds of aptamer for better cell recognition, with deeper understanding of protein distribution on cell membrane. Hopefully, we anticipated our nanoplatform would be further evaluated for the application of CTCs isolation toward other kinds of cancer disease in the future. Finally, we believed that our nanoplatform will further advance the potential application of DNA materials in the field of analytical chemistry and even membrane engineering.

## 4 Experimental Section

##### Assembly and Characterization of the TDNs

All DNA oligonucleotides were purchased from Invitrogen (Thermo Fisher Scientific). Their sequences are listed in Table S1, Supporting Information. All the strands were diluted to 100 µm in TE buffer (10 mmol L^−1^ Tris‐HCl, 1 mmol L^−1^ EDTA, pH 8.0) and quantified by UV before stored at −20 °C. Seven DNA oligonucleotides were equimolarly mixed together in TM buffer (20mm  Tris, 50mm  MgCl_2_, pH 8.0) at final concentration of 1 µm. The mixture was heated at 95 °C  for 5 min, then quickly cooled to 4 °C and maintained for 5 min. The formed DNA tetrahedrons were stored at 4 °C before use. Agarose gel electrophoresis was implemented to confirm the assembly of the DNA tetrahedron. 3% agarose gels were prepared in 1× TBE buffer (89 mmol L^−1^ Tris‐boric, 2 mmol L^−1^ EDTA, pH 8.0) supplemented with YeaRed Nucleic Acid Gel Stain (Yeasen). DNA Samples (10 µL, 1 µm) were mixed with 2 µL 6× loading buffer (Yeasen), run at 100 V for 60 min in 1× TBE, and imaged using a G:Box (Syngene). For AFM imaging of the DNA tetrahedron, fresh mica surface was first incubated with a 2.5 nm solution of the TDN for 5 min. Then, the sample solution was washed off by TM buffer. Finally, 50 µL of the TM buffer was added to the mica surface and AFM imaging was performed under the liquid phase model with a Dimension FastScan (Bruker Santa Barbara, US) and SNL‐10 probes.

##### Cell Culture

Human breast cancer cell lines (MCF‐7, MDA‐MB‐231, and SKBR‐3) were cultured in Dulbecco's modified essential medium (DMEM, Gibco) supplemented with 10% fetal bovine serum (Gibco) and 1% penicillin–streptomycin (Gibco) under 37 °C and 5% CO_2_. Jurkat cells were cultured in the same condition with DMEM replaced by Roswell Park Memorial Institute medium (RPMI‐1640, Gibco).

##### Cellular Distribution of the DNA Nanosynapse

MCF‐7, MDA‐MB‐231, and SK‐BR‐3 were first seeded at a density of 50 000 cells in 35‐mm glass‐bottomed culture dishes. After 12 h, cells were washed by PBS three times, and then incubated with serum‐free DMEM containing Cy3‐labeled bare TDN (Cy3‐labeled strand C) or those with homo/hetero‐multivalent aptamers (TDN‐3 EpCAM or DNA nanosynapse) for 4 h at 37 °C. Free TDN was then washed away by BB buffer (1× PBS, 5 mm MgCl_2_, pH 7.4) for three times. Subsequently, cell membrane was stained with 20 µg mL^−1^ DiO (Beyotime) for 15 min, followed by three times washing by PBS. Cellular distribution of the DNA nanosynapse was imaged by confocal microscope (FV3000 Olympus). For FRET assay, cells were first treated with 200 nm Cy5 and Cy3‐conjugated aptamers, respectively (detailed information could be referred to Figure S4A and Table S1, Supporting Information) for 30 min at 4 °C. After that, cells were washed with PBS and further incubated with 50 nm locking strand for 30 min at room temperature. Cells were washed again with PBS and imaged by CLSM.

##### Cell Capture and Release

MCF‐7, SK‐BR‐3, MDA‐MB‐231 cells were harvested and stained with membrane probe DiO, Dil, and nuclear‐specific dye Hoechst 33 342 (all purchased from Beyotime), respectively, in PBS at 4 °C for 30 min. After three‐time wash by PBS, cells were resuspended in PBS, and cell density was measured by a hemocytometer, except that a small cell number (4–20) was determined as previously reported. A certain number of labeled cells or cell mixtures were spiked with 10^5^ Jurkat cells, and incubated with 200 nm biotin‐labeled TDN‐3 EpCAM, DNA nanosynapse, or cocktail of isotropous TDN‐3 (TDN‐3 EpCAM:TDN‐3 EGFR:TDN‐3 HER2 = 1:1:1) respectively in BB buffer at 4 °C for 30 min with a final volume of 200 µL. 5 µL Dynabeads of 10 mg mL^−1^ were then added and continued incubation for another 30 min. After magnetic separation, cells were washed three times by BB buffer and resuspended in PBS solution. Captured cells were then transferred to a 32‐well plate and counted by fluorescence microscope (IX83, Olympus). The capture efficiency could be denoted as the percentage of captured cells against total cells initially spiked. To capture cells in blood, peripheral blood was first collected from healthy people and handled within 12 h. Blood samples were centrifuged at 800 × *g* for 10 min, and the supernatant of serum was substituted by the same volume of BB buffer. A certain number of DiO‐labeled cells was spiked into the blood and treated as aforementioned. The isolated cells were stained with FITC Anti‐Human CD45 (560 976, BD Biosciences) to count the number of non‐specific WBCs. To achieve cell released, the captured cells were resuspended in DMEM containing 10% FBS, and treated with 0.2 U µL^−1^ DNase I (2212, Takara) at room temperature for 30 min. Next, the mixture was pipetted thoroughly to maximize cell release following magnetic separation of the beads. The released cells were then transferred to a 32‐well plate and counted by fluorescence microscope. Cell released efficiency was calculated as the percentage of released cells to the captured cells.

##### SEM Image of Captured Cells

MCF‐7 cells were incubated with 200 nm biotin‐labeled TDN‐3 EpCAM or DNA nanosynapse in BB buffer at 4 °C for 30 min with a final volume of 200 µL. 5 µL Dynabeads of 10 mg mL^−1^ (65 001, Thermo Fisher Scientific) were then added and continued incubation for another 30 min, followed by magnetic separation. After that, cells were fixed by adding 500 µL glutaraldehyde (2.5%) and incubated overnight at RT. Cells were then washed three times by PBS, and underwent gradient dehydration by exposure to a series of ethanol solutions (30%, 50%, 70%, 90%, 95%, and 100%) for 15 min per turn. Subsequently, cells were treated with 50% HMDS for 15 min, followed by resuspending in 100% HMDS. Cells were added dropwise to silicon wafer and dried in the cupboard overnight. The samples were subjected to gold sputtering and finally imaged by thermal field‐emission environmental scanning electron microscope (Quanta 400F, FEI) at an accelerating voltage of 20 kV.

##### Detection of CTCs in Clinical Samples

Samples were taken from both healthy and breast cancer patients, and handled immediately as described above in the section of cell capture in blood without cell spiking. The detection of CTCs was used by common three‐color immunocytochemistry. To detect CTCs, captured cells were then treated with common three‐color immunocytochemistry. In brief, the captured cells were first fixed with 4% PFA for 15 min, followed by three‐time wash with PBS. Then cells were then permeabilized with 0.1% Triton for 15 min, and washed three times with PBS. After this, cells were blocked with 1% BSA for 40 min, washed with PBS, and stained by PE‐CF594 Anti‐Human Cytokeratin (563 615, BD Biosciences) as well as FITC Anti‐Human CD45 for 30 min. Hoechst 33 342 was added and incubated for another 10 min. Subsequently, the captured cells were washed three times with and resuspended in PBS containing 1% BSA. The captured cells were imaged in fluorescence microscope. Cells with a staining profile of Hoechst 33 342^+^/CD45^−^/CK^+^ and proper morphology were scored as CTCs. The study was performed in compliance with the relevant laws and institutional guidelines, and was approved by the Institutional Review Board (IRB) of Sun Yat‐sen University (IRB approval 2019‐L054‐1). All patients gave written informed consent.

##### FISH in Captured CTCs from Patients

Captured cells were transferred onto a poly‐L‐lysine‐coated glass slide and air‐dried overnight before further processing. Centromere‐Specific Probe 17 and gene‐specific probes for HER‐2 were purchased from GP Medical Technologies, Ltd. (Beijing). Hybridization and post‐hybridization washes were performed following manufacturer's protocols. After DAPI (Beyontime) staining, the slides were mounted in antifade solution (Solarbio) and examined by fluorescence microscope.

##### Cell Viability Assay

The captured cells were stained with calcein‐AM (Beyotime) according to the manufacturer's protocol. After this, cells were imaged by fluorescence microscope. The calcein‐AM+ cells were denoted as live cells.

##### Culture of the Captured Cells

After magnetic isolation, cells were resuspended in DMEM containing 20% FBS and 1% penicillin−streptomycin solution, followed by culture in 96‐well plate under 37 °C and 5% CO_2_. At day 2, each well was washed with PBS and filled with fresh DMEM supplemented with 10% FBS and 1% penicillin−streptomycin solution. The growth of the cells was imaged by bright‐field microscope.

##### Statistical Analysis

Graphic presentation was performed using GraphPad Prism Software 8. The sample size for each group was at least three and data were expressed as mean ± SD. The yield of the assembled DNA nanosynapse, as well as the counting of the capture cells were analyzed by ImageJ software.

## Conflict of Interest

The authors declare no conflict of interest.

## Supporting information

Supporting Information
