# Supplementary material for: Bioinspired DNA Nanointerface with Anisotropic Aptamers for Accurate Capture of Circulating Tumor Cells
Source: Adv Sci (Weinh). 2020 Aug 9;7(19):2000647. doi: 10.1002/advs.202000647 (PMC7539197; doi:10.1002/advs.202000647)
Supplement: Supplementary file 1 — Supporting Information [file ADVS-7-2000647-s001.pdf]

© 2020 Wiley-VCH GmbH

ADVANCED SCIENCE
Open Access

Supporting Information

for *Adv. Sci.,* DOI: 10.1002/advs.202000647

Bioinspired DNA Nanointerface with Anisotropic Aptamers for Accurate Capture of Circulating Tumor Cells

Weiwei Qin, Liang Chen, Zhiru Wang, Qian Li, Chunhai Fan, Minhao Wu, and Yuanqing Zhang**

Copyright WILEY-VCH Verlag GmbH & Co. KGaA, 69469 Weinheim, Germany, 2020.

Supporting Information

Bioinspired DNA Nanointerface with Anisotropic Aptamers for Accurate Capture of Circulating Tumor Cells

Weiwei Qin, Liang Chen, Zhiru Wang, Qian Li, Chunhai Fan, Minhao Wu, and Yuanqing Zhang**

Dr. W. Qin, L. Chen, Z. Wang, Pro. Y. Zhang
Guangdong Key Laboratory of Chiral Molecule and Drug Discovery
School of Pharmaceutical Sciences
Sun Yat-sen University
Guangzhou, Guangdong 510006, China
Email: zhangyq65@mail.sysu.edu.cn

Dr. W. Qin
College of Materials and Energy
South China Agricultural University
Guangzhou, Guangdong 510642, China

Dr. Q. Li, Pro. C. Fan
School of Chemistry and Chemical Engineering
Shanghai Jiao Tong University
Shanghai 200240, China
Email: liqian2018@sjtu.edu.cn

Pro. M. Wu
Zhongshan School of Medicine
Sun Yat-sen University
Guangzhou 510080, China

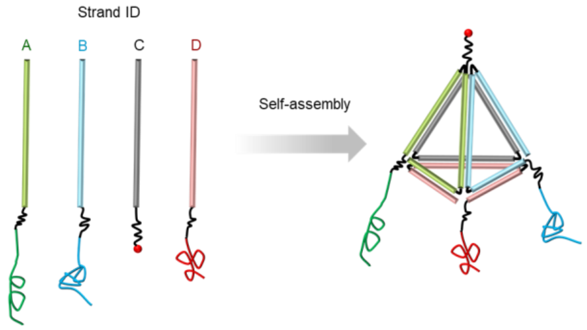

Figure S1. Schematic map illustrating the assembly of the DNA nanosynapse. Green, blue, grey and red lines represent strand A, B, C and D in Table S1 respectively. The three protruded strands are denoted as three differet aptamers aganist EpCAM, HER2 and EGFR. The red dot in strand C represents Cy3 or biotin. For the assembly of TDN 3 EpCAM (HER2/EGRF), Strand A-S1, B-S1, C-biotin, D-S1 and EpCAM-S1c (HER2-S1c/EGRF-S1c) were used. For the assembly of DNA nanosynapse, Strand A-S1, B-S2, C-biotin, D-S3, EpCAM-S1c, HER2-S2c, and EGFR-S3c were used.

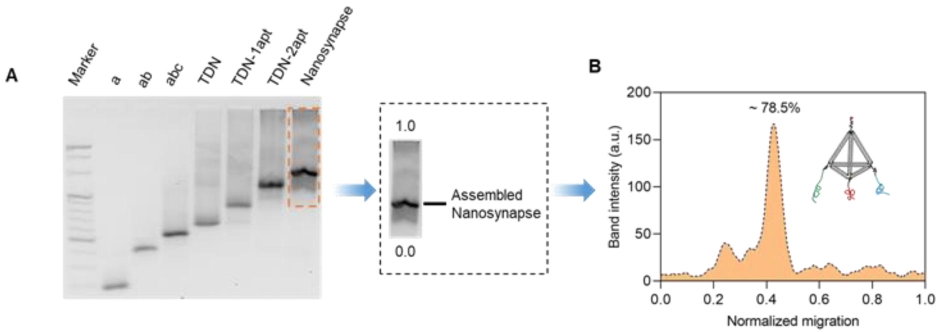

Figure S2. (A) Agarose gel electrophoresis result of the DNA nanosynapse. The dash area indicated the cropped image subjected to the following intensity analysis. (B) The yield (~78.5%) of DNA nanosynapse from agarose gel electrophoresis after analysis by Image J.

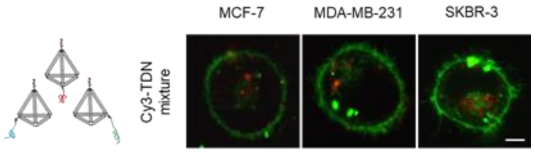

Figure S3. Cellular distribution of the DNA nanostructure mixture with separately conjugated aptamers. Confocal laser scanning microscopy of three different cell lines incubated with DNA nanostructure mixture for 4 h. The nanostructures were labeled with Cy3 (Red) and cell membrane was stained with DiO (green). Scale bar: 5 µm.

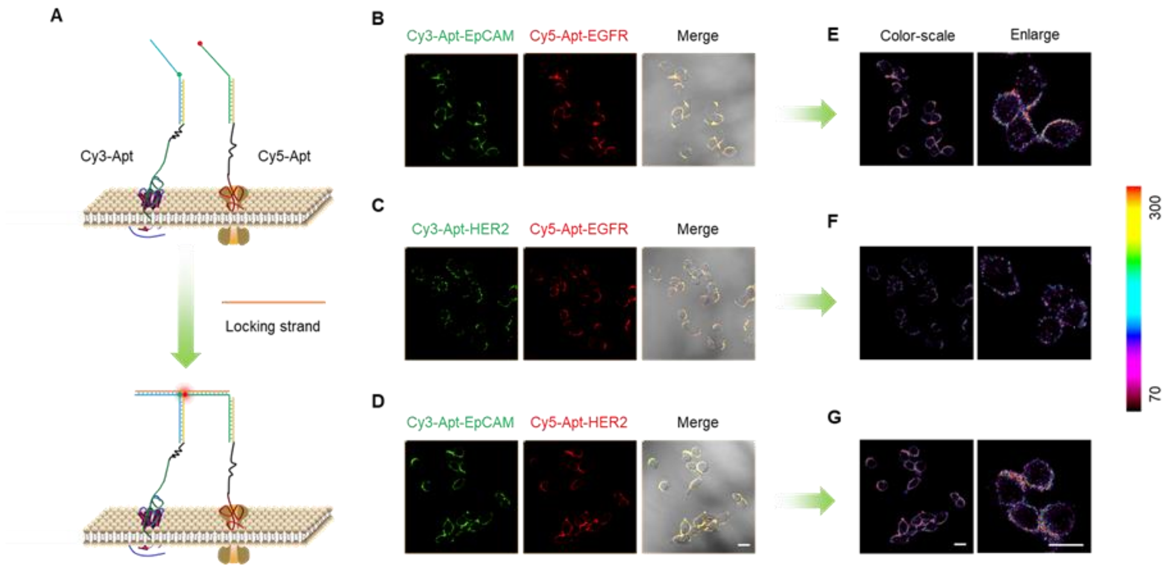

Figure S4. Recruitment of Cy3- and Cy5-conjugated aptamers on living cell membrane. (A) Schematic illustration of locking-strand-induced protein recruitment on the cell membrane. Apt-EpCAM (or Apt-HER2, or Apt-EGFR) was conjugated with Cy5-S2-S1, and Apt-EGFR (or Apt-EGRF, or Apt HER2) was conjugated with S3-Cy3-S1. Sequence of all the strands were listed in Table S1. (B-D) CLSM images of MDA-MB-231 cells bound with fluorescence-conjugated aptamers, flollowing treated with locking strand. Before photobleaching, cell membranes showed FRET-induced Cy5 fluorescence signal. Green channel: excitation 543 nm/emission 560−610 nm. Red channel: excitation 543 nm/emission 650LP filter. (E-G) Color-scale images showing the enhancement of Cy3 emission after receptor Cy5 photobleaching with 640 nm laser. Scale bar: 20 μm.

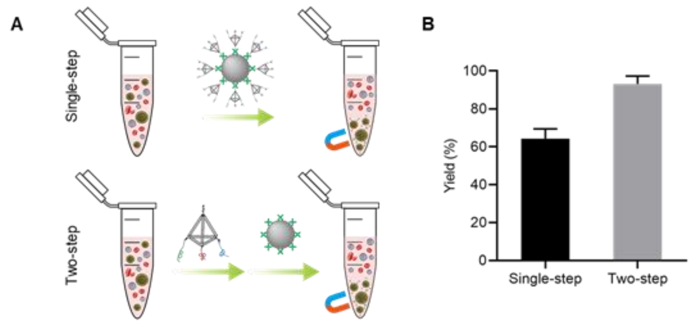

Figure S5. Comparison of capture efficiency by two different strategies. (A) Schematic representation of two different strategies for CTC isolation. In single-step strategy, the DNA nanostructures are conjugated to the magnetic beads prior to incubation with cells, while in two-step strategy, the DNA nanostructures and magnetic beads were incubated with cells successively. (B) Capture efficiency of two different strategies.

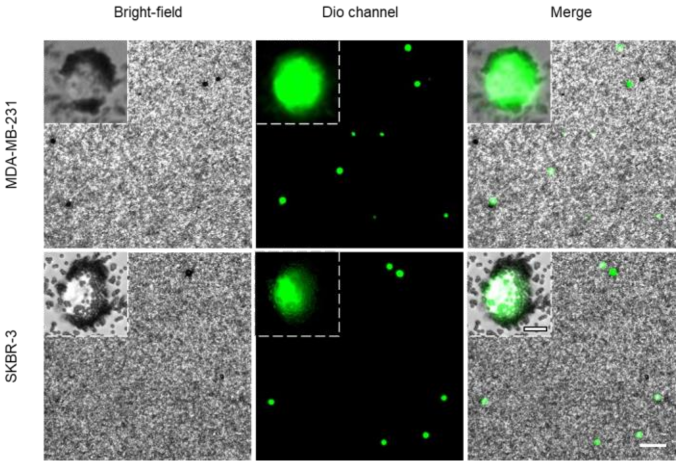

Figure S6. Fluorescence microscopy images of the captured MDA-MB-231 and SKBR-3 cells labeled with DiO (green). Scale bar: 100 μm; Insect: 5 μm.

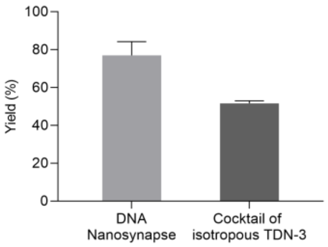

Figure S7. The capture performance towards 1000 MDA-MB-231 cells of DNA nanosynapse, and that of the cocktail of TDN-3 EpCAM, TDN-3 EGFR, and TDN-3 HER2.

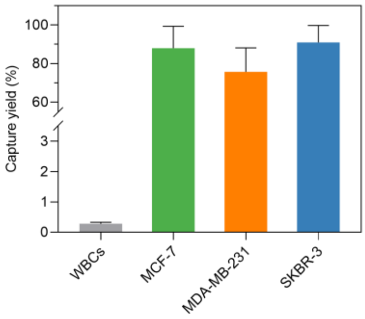

Figure S8. Cell capture yield by DNA nanosynapse towards WBCs and three different cell lines of 1000 cells spiked in whole blood sample from healthy donors.

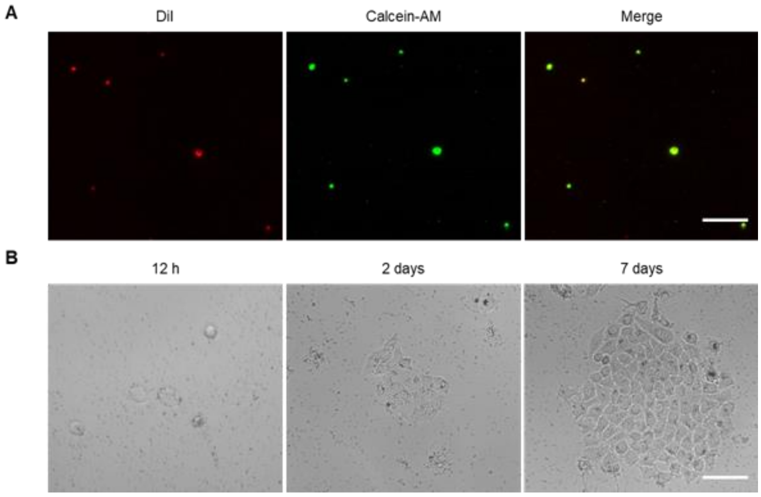

Figure S9. Cell viability analysis of the captured MCF-7 cells. (A) Fluorescence microscopy images of the captured MCF-7 cells labeled with Dil (red). Live cells are stained with Calcein-AM (green). (B) Bright-field microscopy images of the captured MCF-7 cells that cultured in 96-well plates after 0.5, 2, and 7 days. Scale bar: 200 μm (A) and 100 μm (B).

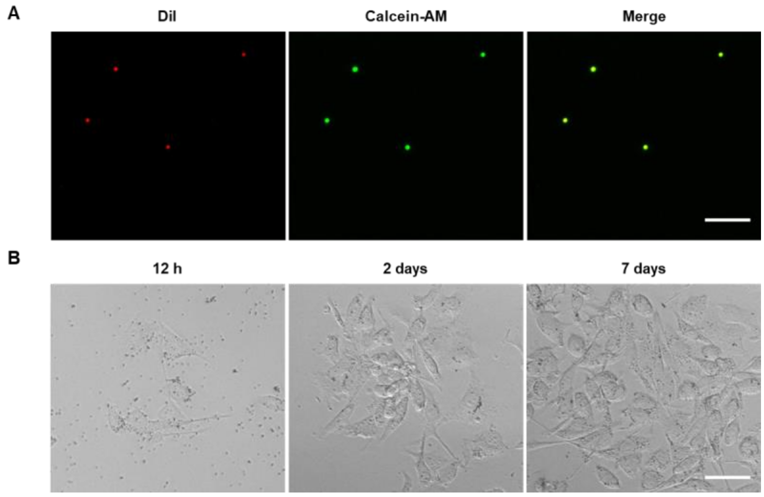

Figure S10. Cell viability analysis of the captured MDA-MB-231 cells. (A) Fluorescence microscopy images of the captured MDA-MB-231 cells labeled with Dil (red). Live cells are stained with Calcein-AM (green). (B) Bright-field microscopy images of the captured MCF-7 cells that cultured in 96-well plates after 0.5, 2, and 7 days. Scale bar: 200 μm (A) and 100 μm (B).

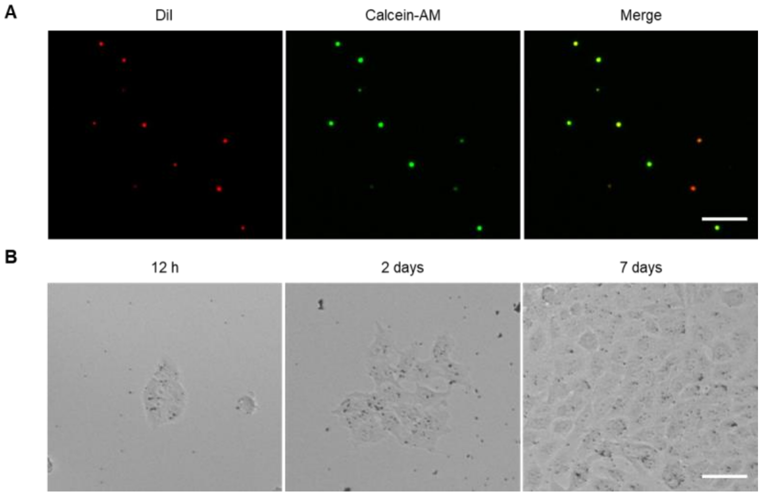

Figure S11. Cell viability analysis of the captured SKBR-3 cells. (A) Fluorescence microscopy images of the captured SKBR-3 cells labeled with Dil (red). Live cells are stained with Calcein-AM (green). (B) Bright-field microscopy images of the captured MCF-7 cells that cultured in 96-well plates after 0.5, 2, and 7 days. Scale bar: 200 μm (A) and 100 μm (B).

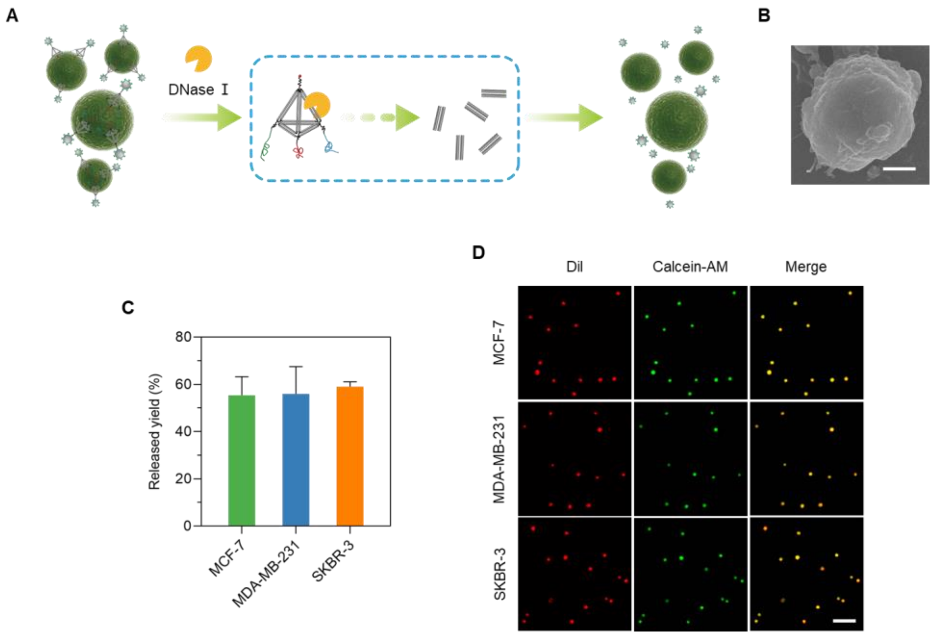

Figure S12. (A) Schematic illustrating the strategy used for the released of capture CTCs. (B) SEM image of the released MCF-7 cells. Scale bar: 2 μm. (C) The enzymatic released efficiency of the captured MCF-7, MDA-MB-231 and SKBR-3 cells, respectively. The total cell numbers in the buffer was 1000 cells/mL. (D) Fluorescence microscopy imaging results demonstrated high viability of recovered cells. Cells are labeled with Dil (red) before cell spiking and then stained with Calcein-AM (green) after recovery. Scale bar: 100 μm.

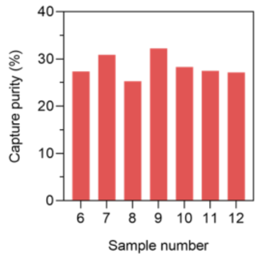

Figure S13. Purity of isolated CTCs from breast cancer patients (6-12). The purity was denoted as the percentage of CTCs to total captured cells.

Table S1. DNA sequences for the assembly of DNA nanostructures in this work.

ID	Sequence（5'-3'）
A	ACATTCCTAAGTCTGAAACATTACAGCTTGCTACACGAGAAGAG CCGCCATAGTA
B	TATCACCAGGCAGTTGACAGTGTAGCAAGCTGTAATAGATGCGA GGGTCCAATAC
C	TCAACTGCCTGGTGATAAAACGACACTACGTGGGAATCTACTAT GGCGGCTCTTC
D	TTCAGACTTAGGAATGTGCTTCCCACGTAGTGTCGTTTGTATTGG ACCCTCGCAT
Cy3-C	Cy3- TCAACTGCCTGGTGATAAAACGACACTACGTGGGAATCTACTAT GGCGGCTCTTC
C-(peg)3- biotin	biotin-(peg)3- TCAACTGCCTGGTGATAAAACGACACTACGTGGGAATCTACTAT GGCGGCTCTTC
A-S1	ACATTCCTAAGTCTGAAACATTACAGCTTGCTACACGAGAAGAG CCGCCATAGTACTGACCACGAGCTCCATTAC
B-S1	TATCACCAGGCAGTTGACAGTGTAGCAAGCTGTAATAGATGCGA GGGTCCAATACCTGACCACGAGCTCCATTAC
D-S1	TTCAGACTTAGGAATGTGCTTCCCACGTAGTGTCGTTTGTATTGG ACCCTCGCATCTGACCACGAGCTCCATTAC
B-S2	TATCACCAGGCAGTTGACAGTGTAGCAAGCTGTAATAGATGCGA GGGTCCAATACCATCCATTCCAGTGAGAGAT
D-S3	TTCAGACTTAGGAATGTGCTTCCCACGTAGTGTCGTTTGTATTGG ACCCTCGCATTACGAGAAGAGAATCCTGAA
EpCAM-S1c (Apt-EpCAM)	CACTACAGAGGTTGCGTCTGTCCCACGTTGTCATGGGGGGTTGG CCTGTTTTTGTAATGGAGCTCGTGGTCAG
HER2-S2c	AACCGCCCAAATCCCTAAGAGTCTGCACTTGTCATTTTGTATATG TATTTGGTTTTTGGCTCTCACAGACACACTACACACGCACATTTT TATCTCTCACTGGAATGGATG
EGFR-S3c	TACCAGTGCGATGCTCAGTGCCGTTTCTTCTCTTTCGCTTTTTTT GCTTTTGAGCATGCTGACGCATTCGGTTGACTTTTTTTCAGGATT

	CTCTTCTCGTA
HER2-S1c (Apt-HER2)	AACCGCCCAAATCCCTAAGAGTCTGCACTTGTCATTTTGTATATGTATTTGGTTTTTGGCTCTCACAGACACACTACACACGCACATTTTTGTAATGGAGCTCGTGGTCAG
EGFR-S1c (Apt-EGFR)	TACCAGTGCGATGCTCAGTGCCGTTTCTTCTCTTTCGCTTTTTTTGCTTTTGAGCATGCTGACGCATTCGGTTGACTTTTTGTAATGGAGCTCGTGGTCAG
Cy5-S2-S1	Cy5-TCACCATTCCAGTGAGAGATCTGACCACGAGCTCCATTAC
S3-Cy3-S1	TACGAGAAGAGAATCCTGA/iCy3dT/CTGACCACGAGCTCCATTAC
Locking strand	ATCTCTCACTGGAATGGTGAATCAGGATTCTCTTCTCGTA

Table S2. Comparison between current status of EpCAM-based isolation and anisotropic aptamer-based capture against low EpCAM cells in this work.

Low EpCAM cell line	Capture ligand	Capture efficiency	Reference
T24 cells<br>Colo-320 cells	EpCAM antibody (CellSearch)	2%	*Sci. Rep.* 2015, 5, 12270
MDA-MB-231 cells	EpCAM aptamer	16%	*J. Am. Chem. Soc.* 2017, 139, 2741−2749
	EpCAM antibody (CellSearch)	< 10%	*Nat. Nanotech.* 2017, 12, 274–281
	EpCAM antibody	60 %	*Adv. Funct. Mater.* 2019, 29, 1808961
	anisotropic aptamer	77%	This work

Table S3. The differences in capture performance between previous work and this work.

<table>
<tr><th rowspan="2">Reference</th><th rowspan="2">DNA nanostructures</th><th colspan="2">Capture efficiency</th><th rowspan="2">Buffer for cell spiking</th></tr>
<tr><th>MCF-7 cells</th><th>MDA-MB-231 cells</th></tr>
<tr><td>J. Am. Chem. Soc. 2019, 141, 47, 18910–18915</td><td rowspan="2">TND-3 EpCAMs</td><td>90.5%</td><td>75%</td><td>Pure PBS</td></tr>
<tr><td rowspan="2">This work</td><td>93.1%</td><td>29.8%</td><td rowspan="2">PBS containing 10^5 Jurkat cells</td></tr>
<tr><td>DNA nanosynapse</td><td>91.1%</td><td>77%</td></tr>
</table>
